# Comparison of Narrow (<3.75 mm) and Standard (≥3.75 mm) Diameter Implants Supporting the Same Multiple Fixed Prostheses and Mirroring Real-World Clinical Scenarios: Non-Randomized Clinical Trial

**DOI:** 10.3390/dj13090420

**Published:** 2025-09-12

**Authors:** Eduardo Anitua, Ander Alcaine, Mohammad Hamdan Alkhraisat

**Affiliations:** 1 University Institute for Regenerative Medicine and Oral Implantology—UIRMI (UPV/EHU-Fundación Eduardo Anitua), 01007 Vitoria, Spain; mohammad.hamdan@bti-implant.es; 2 BTI Biotechnology Institute, 01007 Vitoria, Spain; 3 Department of Cellular Biology and Histology, Faculty of Medicine and Nursing, Universidad del País Vasco/Euskal Herriko Unibertsitaea (UPV/EHU), 48940 Leioa, Spain; a.alcaine@hotmail.com; 4 Oral and Maxillofacial Surgery, Oral Medicine and Periodontics Department, Faculty of Dentistry, University of Jordan, Amman 11942, Jordan

**Keywords:** dental implant, narrow dental implants, marginal bone loss, implant survival, fixed prothesis

## Abstract

**Objective:** To compare the survival of narrow (<3.75 mm) implants and standard diameter (≥3.75 mm) implants supporting the same multiple fixed prostheses and mirroring real-world clinical scenarios. **Methods:** This is a controlled clinical trial where both test (diameter < 3.75 mm) and control (diameter ≥ 3.75 mm) implants supported the same prosthesis. The principal variable was implant survival and the secondary variables included demographic, surgical and prosthetic variables. Statistical analyses were conducted to compare these variables between the study groups. **Results:** A total of 42 patients participated in this study, with an age range of 39 to 92 years. The follow-up period was 36 months. Narrow diameter implants (NDIs) were predominantly placed in the premolar region and more frequently in bone types I and II compared to wider diameter implants. No implant failures were recorded during the study period. Marginal bone level remodeling showed statistically significant differences between the study groups at 12-month follow-up. However, these differences were no longer significant after 3 years of follow-up (Test: median −0.2 mm, range −1.5 to 0.8 mm; Control: median 0.0 mm, range −1.3 to 0.8 mm; *p* = 0.119). None of the prostheses failed, and all remained free of technical complications throughout the study. **Conclusions:** Within the limitations of this study, narrow-diameter implants demonstrated comparable clinical outcomes to standard-diameter implants when supporting the same prostheses.

## 1. Introduction

The three-dimensional atrophy of the alveolar bone, provoked by edentulism, affects the stability and function of conventional prosthesis [1,2,3,4]. Moreover, it may restrict the use of dental implants without the performance of an alveolar bone augmentation procedure [5]. In 2004, a comfort zone for the positioning of the dental implants was defined by the ITI consensus to ensure optimal implant position and esthetic outcomes [5,6]. The presence of 1 mm of buccal bone around the dental implants in posterior regions would provide long-term stability [7]. In the presence of alveolar bone atrophy, optimal positioning of regular-diameter implants may not be feasible without bone augmentation [5]. Clinicians may opt for narrow-diameter implants (diameter < 3.75 mm) when factors such as age, medical conditions, and patient willingness, among others, constrain the feasibility of bone augmentation procedures. NDIs have several indications, such as the replacement of mandibular incisors and maxillary lateral incisors, reduced interdental space (<6 mm), and narrow alveolar bone width (<5 mm) [8].

Narrow-diameter implants (NDIs) have evolved from their initial role as transitional implants to becoming reliable options for definitive support of single crowns, partial prostheses, and complete prostheses across both anterior and posterior regions of the maxilla and mandible [9,10,11,12,13,14]. Due to their smaller size, NDIs possess a reduced surface area for osseointegration and exhibit lower load resistance compared to wider implants [15,16]. Although some studies highlight the narrow diameter of the implants as a potential risk factor for fractures [17,18,19], particularly in posterior locations and single restorations, it is crucial to understand that implant fracture is multifaceted. Factors such as patient characteristics, the design of the implant, prosthesis configuration, and marginal bone loss all contribute to the risk of implant fracture [17].

The current available evidence supports that NDIs reduce the need for alveolar ridge augmentation and thus the number of surgical procedures to achieve successful outcomes [11,13]. When compared to regular-diameter implants, NDIs have achieved higher patient satisfaction and lower treatment costs [20]. As compared to one-piece implants, two-piece NDIs offer flexibility to adapt to possible changes in the peri-implant tissues over time [9].

Real-world settings are relevant to the representation of the real-world clinical scenario that may be idealized in the context of a randomized clinical trial [21]. Assessing NDIs in a real-world scenario and under the same prosthesis as the regular diameter implants are gaps that this study aims to bridge. The objective of this study is to evaluate the survival of narrow dental implants (<3.75 mm) in multiple fixed prostheses in comparison with standard diameter dental implants (≥3.75 mm). The null hypothesis of the study was that narrow dental implants under the evaluated conditions have the same survival rate and clinical performance as standard diameter implants.

## 2. Materials and Methods

### 2.1. Trial Design

This is a non-randomized controlled clinical trial where both test (diameter < 3.75 mm) and control (diameter ≥ 3.75 mm) implants supported the same prosthesis. This approach enabled paired analysis by comparing intra-subject performance, helping to minimize confounding factors associated with biological variability. No randomization process was carried out, and the selection of implants was performed by the surgeon following the daily clinical practice. The clinical trial is registered in ClinicalTrials.gov under the ID: NCT04006782.

### 2.2. Ethical Considerations

The study adhered to the ethical principles of the Declaration of Helsinki and was approved by the Research Ethical Committee of University Hospital of Álava (FIBEA-02-EP/19/Implantes estrechos). Prior to participation, all individuals provided written informed consent, confirming their voluntary involvement and understanding of the study’s procedures, potential risks, and benefits. The trial is ongoing, and this interim report presents the 1- and 3-year outcomes. 

### 2.3. Participants

Patients were recruited at a private clinical center in Vitoria, Spain, between May 2019 and July 2021.

#### 2.3.1. Inclusion Criteria

- Patients of legal age (>18 years) of both sexes.
- Clinical need for multiple fixed alveolar ridge rehabilitations.
- Clinical suitability to insert, at least, one narrow dental implant splinted to a standard diameter dental implant.
- Signed informed consent.

#### 2.3.2. Exclusion Criteria

- Presence of an active infection.
- Being under active treatment with, or having received in the last 30 days, treatment with radiotherapy, chemotherapy, immunosuppressants, systemic corticosteroids, and/or anticoagulants.
- Presence of severe hematologic disorders.
- Chronic treatment with non-steroidal anti-inflammatory drugs (NSAIDs) or other anti-inflammatory drugs.
- Previous diagnosis of chronic hepatitis or liver cirrhosis.
- Presence of Diabetes mellitus with improper metabolic control (glycosylated hemoglobin higher than 9%).
- Patients subjected to dialysis.
- Presence of malignant tumors, hemangioma, or angioma in the surgical area.
- History of ischemic cardiopathy in the last year.
- Pregnancy or plan to become pregnant during the study.
- Metabolic bone disease
- Patient receiving treatment with oral or intravenous bisphosphonates.
- Any other condition incompatible with participation in the study.

### 2.4. Randomization and Blinding

No randomization was performed. The selection of the implant to the anatomical site was decided by the clinician considering the daily clinical practice. According to clinical practice, a narrow implant was placed in cases where the alveolar ridge was too narrow to accommodate a regular-diameter implant.

Given the characteristics of the study, no blinding technique was planned for the researchers. The data collection notebook included only the patient number as the sole identifying detail. The correspondence between identifying data and patient numbers was kept in a document safeguarded by the principal investigator and was not accessible to the researchers analyzing the study variables.

### 2.5. Study Groups

In the experimental group, narrow implants (diameter < 3.75 mm) INTERNA^®^ (UnicCA^®^, BTI Biotechnology Institute, Vitoria, Spain) with variable lengths (6.5 to 10 mm) and an internal connection were studied. The control group consisted of the same implant with a regular diameter (diameter ≥ 3.75 mm). No bone augmentation was performed to permit the insertion of the dental implants.

### 2.6. Study Variables

#### 2.6.1. Principal Variable

Implant survival referred to the physical presence of the implant in the patient’s mouth at the time of evaluation. An implant was considered a failure if it was not present in the mouth at the time of evaluation or if its removal was deemed necessary based on clinical criteria like excessive mobility and severe peri-implant disease.

#### 2.6.2. Secondary Variables

Demographic variables (sex, age), medical history, and lifestyle factors such as smoking and alcohol consumption, implant location, type of antagonist, bone type, insertion torque, type of prosthesis (partial or complete), and the length of dental implants. Secondary efficacy outcomes include marginal bone loss, prosthesis survival, and technical complications such as porcelain fracture, screw loosening, screw fracture, and prosthesis fracture.

Marginal bone loss refers to the change in the marginal bone level at implant loading and at the follow-up times (1 and 3 years). Marginal bone level was defined as the distance between the implant platform and the most coronal bone-to-implant contact point. This distance was measured in millimeters on both the mesial and distal sides of the implant using periapical radiographs. These were obtained using the Sirona intraoral radiographic system (Dentsply Sirona, Bensheim, Germany). Radiographs were acquired using the parallel technique with a Rinn XPC-ORA radiographic positioner (Dentsply Sirona, Charlotte, NC, USA). Periapical radiographs were visualized in a DIGORA^®^ for Windows software (version 2.9.113.490, Soredex, Milwaukee, WI, USA), and the known implant length was used to calibrate the measurements. A calibrated researcher performed all the measurements.

### 2.7. Interventions

Before surgery, 2 g of amoxicillin or 600 mg of clindamycin (allergy to penicillin) and 1 g of paracetamol 60 min were given to the patient before surgery as prophylactic medication. All patients underwent local anesthesia (4% articaine hydrochloride with epinephrine 1:100,000 (Artinibsa, Laboratorios Inibsa, Barcelona, Spain)), followed by the elevation of a full-thickness flap to facilitate implant placement. The implants utilized in this study featured an identical surface treatment (UnicCa^®^ surface, BTI Biotechnology Institute, Vitoria, Spain). A low-speed drilling technique was employed to prepare the implant bed [22]. The implants were initially inserted by the same surgeon (E.A.) using a surgical motor set at 25 Ncm. They were subsequently seated at the alveolar bone crest level with a calibrated torque wrench. Insertion torque and bone type at the site were documented. Implants were immediately loaded if the insertion torque was ≥25 Ncm and the bone type was classified as I, II, or III. In such cases, an intermediate abutment was screw-connected to the dental implant, and a provisional metal-resin veneered fixed partial denture (FPD) was placed. Otherwise, a covering screw was placed for submerged healing. In every case, liquid plasma rich in growth factors (PRGF) was applied to the implant socket before inserting the implant. The PRGF was prepared following the manufacturer’s instructions (KMU 15, BTI Biotechnology Institute, Vitoria, Spain) [23,24]. Briefly, 9 mL tubes containing 3.8% sodium citrate were used to extract the blood. After centrifugation, the plasma column was fractioned into F2 and F1. F2 contains 2 mL of the plasma column just above the buffy coat, and F1 contains the plasma above the F2.

Oral hygiene instructions (soft cleaning of the surgical area) were prescribed. Paracetamol up to 1 g every 8 h was prescribed as an analgesic. If necessary, it may be combined with metamizole 575 mg, 1 or 2 capsules every 8 h.

Patients undergoing delayed loading required a second surgical procedure to remove the covering screw and connect the definitive abutment.

Regardless of whether immediate or delayed loading was performed, the same type of definitive intermediate abutments was used (Multi-IM^®^ transepithelial abutments; BTI Biotechnology Institute, Vitoria, Spain). For loading, the intermediate abutment was connected to the implant. The impression-making and prosthetic rehabilitation were conducted at the gingival level to facilitate the delivery of a screw-retained prosthesis. For the provisional phase, a resin-veneered articulated titanium bar system (BTI Biotechnology Institute, Vitoria, Spain) was utilized. The definitive prosthesis featured a computer-designed and computer-manufactured metallic framework, which was subsequently veneered with ceramic to achieve the final screw-retained restoration in a mutually protected occlusion.

### 2.8. Sample Size Calculation

For the sample size calculation in this study, the estimate was based on the meta-analysis published by Schiegnitz et al. [25]. Considering the mean risk ratio of 2.54 obtained in the meta-analysis (narrow implants compared to regular-diameter implants), a recruitment period of 1.5 years, an average survival time of 5 years, and a dropout rate of 15%, it was necessary to include 58 narrow-diameter implants and 58 regular-diameter implants.

This sample size was required to reject the null hypothesis—that the survival curves of NDIs and regular-diameter implants are equal—with a probability of 0.8. The Type I error rate was set at 0.05.

### 2.9. Statistical Methods

A descriptive analysis of the sample was performed based on demographic variables (age and sex) and clinical characteristics (dental and medical history). For quantitative variables, the median and range were calculated. The normality of the data distribution was assessed using the Shapiro–Wilk test, which informed the choice between the paired Student’s *t*-test or the non-parametric Wilcoxon signed-rank test, as appropriate. In the case of uneven sample size between the groups, the Welch *U* test was used. Qualitative variables were analyzed using frequency distributions and compared using the chi-square test. Then, a multiple regression analysis was performed to assess the influence of baseline differences on the variation in marginal bone level. For that, the association between the mean variation in marginal bone level, study group, jaw, bone type, insertion torque, loading protocol, and antagonist was assessed by Spearman’s correlation test. Then, factors with significant Spearman correlation were introduced in the multiple regression analysis model or ANOVA. All statistical analyses were conducted using IBM SPSS Statistics software (Version 15, SPSS Inc., Chicago, IL, USA), with statistical significance set at *p* < 0.05.

### 2.10. Report/Publication Guidelines

This article was written following the CONSORT guidelines [26].

## 3. Results

**Figure 1:**
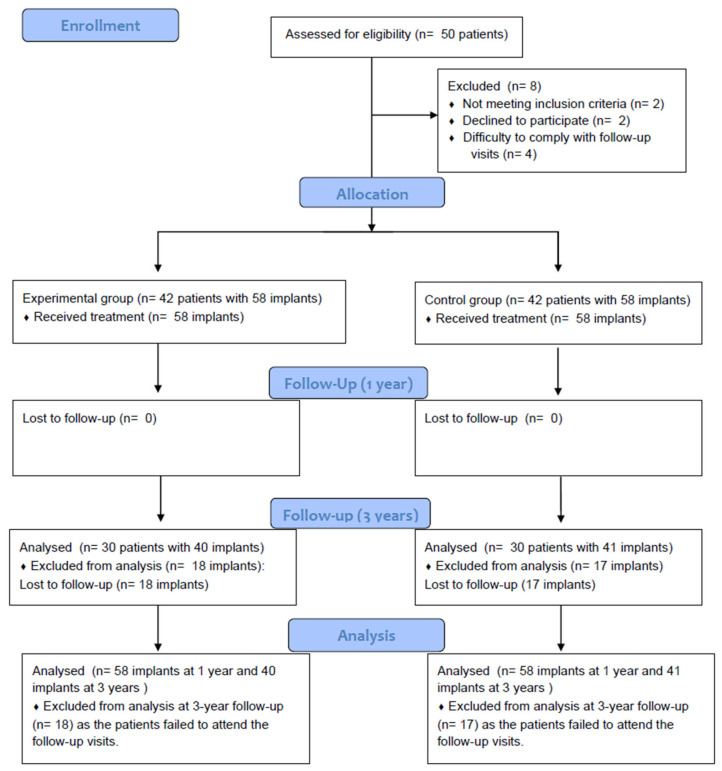
CONSORT flow diagram.

Fifty patients were screened for eligibility, and 42 were included (Figure 1). The reasons for exclusion were non-meeting of the inclusion criteria (two patients), decline to participate (two patients), and difficulty complying with the scheduled follow-up visits (four patients). During the follow-up, 12 patients failed to attend the 3-year follow-up visit. The analysis was performed for 58 implants in each group at 1-year follow-up. At 3-year follow-up, the test group had 40 implants and the control group 41 implants (Figure 1). One lost patient had two test implants and one control implant.

The participants had an age range of 39 to 92 years (mean and standard deviation of 65 and 9 years, respectively. In total, 24 patients were females and 18 were males. Among them, four patients were smokers (2 to 15 cigarettes/day), and only two indicated alcohol consumption (10 to 20 g of alcohol). The majority (71.4%, 30 patients) reported no significant medical history. Among those with pre-existing conditions, hypertension (two patients), hiatal hernia (two patients), allergies (two patients), appendicitis (one patient), breast cancer, varicose veins, cataracts (one patient combined), hypothyroidism, sleep disorders (one patient combined), hysterectomy (one patient), migraines, trochanteritis (one patient), and prostate conditions (one patient) were reported. The analysis of dental history in 42 patients revealed parafunction was the predominant condition, present in 81% of patients with reported dental issues, with moderate bruxism being most frequent (31 cases, 73.8% of the total cohort). Associated findings included tooth wear (69% of affected patients), cracks/fissures (64.3%), and parafunctional habits (59.5%). Comorbidities included sleep breathing disorders (28.6% with snoring/oral breathing) and previous TMJ pathology (9.5%). Severe bruxism with significant tooth wear and articular noises was less common (4.8%). One case (2.4%) reported previous periodontal disease alongside moderate bruxism. The cumulative distribution shows these conditions frequently coexisted, with 61.9% of affected patients presenting at least three concurrent findings (bruxism + wear + fissures + parafunction).

The implants were mainly placed in the mandible for both groups, with premolars and molars being the most frequent locations for NDIs and regular-diameter implants, respectively (Table 1).

**Figure 2:**
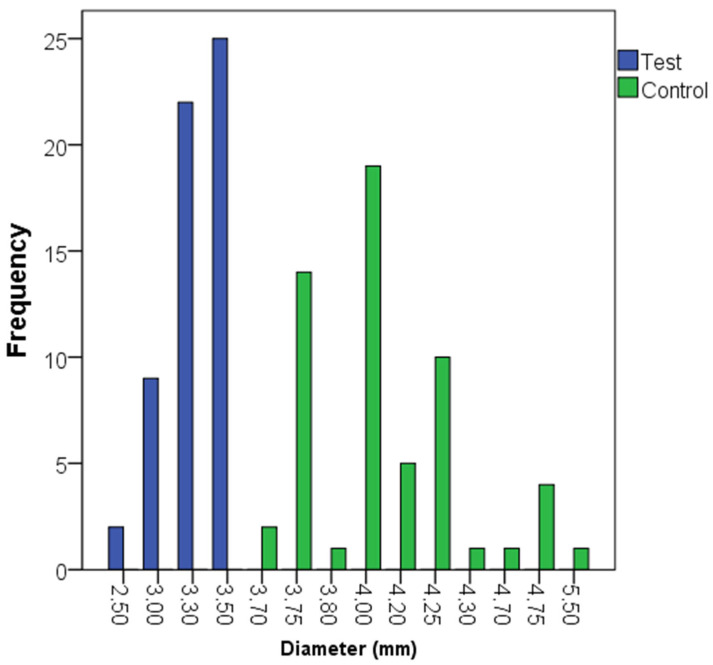
Diameter of the dental implants in the test (narrow implants) and control (regular implants) groups.

**Figure 3:**
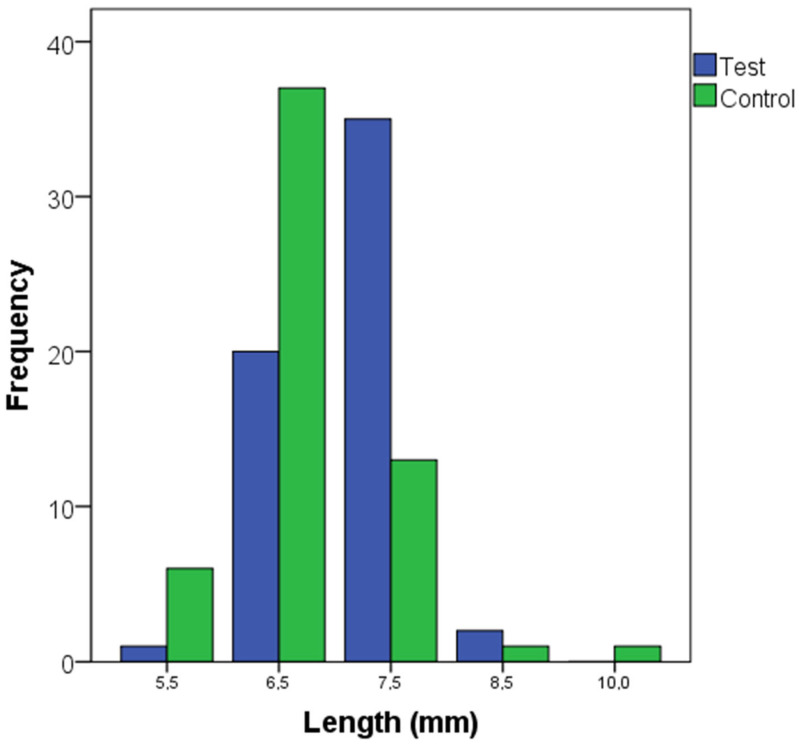
Length of the dental implants in the test (narrow implants) and control (regular implants) groups.

The distribution of the lengths and diameters of the dental implants is described in Figure 2 and Figure 3. The most frequent implant lengths were 6.5 and 7.5 mm in the control and test groups, respectively. The baseline characteristics between the test and control groups are described in Table 2, including bone type, insertion torque, and loading protocol. Bone quality was categorized into Types I to IV, with the test group showing more Type I cases and fewer Type IV cases compared to the control group. Insertion torque was similar between groups, with median values of 60 Ncm for both groups (*p* = 0.888). Loading protocols (immediate and delayed) were also balanced, with no significant differences between the study groups (*p* = 1.000).

Regarding prosthetic parameters, Table 3 shows that the distribution of antagonist types (implant, tooth, or removable denture) was similar between groups (*p* = 0.752). There were 57 partial fixed prostheses and only 1 complete fixed prosthesis. All the prostheses were screw retained to the intermediate abutment. No prosthesis failures were reported at either the 12- or 36-month follow-ups.

Marginal bone level (MBL) remodeling showed statistically significant differences between the study groups at 12-month follow-up (Table 3). Spearman’s correlation analysis indicated the presence of a significant association between mean marginal bone variation at 1 year with study group (*p* = 0.028). The study group was associated with bone type (0.007). The ANOVA analysis indicated that neither the study group (*p* = 0.290) nor the bone type (*p* = 0.499) had a significant effect on the mean variation in the marginal bone level at 1 year.

However, these differences were no longer significant after 3 years of follow-up (Test group: median −0.2 mm, range −1.5 to 0.8 mm; Control group: median 0.0 mm, range −1.3 to 0.8 mm; *p* = 0.079). Spearman’s correlation analysis indicated the presence of a significant association between mean marginal bone variation at 3 years with jaw type (*p* = 0.015). Jaw type was associated with bone type (*p* = 0.000), insertion torque (*p* = 0.000), and loading protocol (*p* = 0.003). The multiple regression analysis indicated the absence of effect of any of these factors on the variation in marginal bone level at 3 years.

The prostheses in both the test and control groups remained free of technical complications throughout the study. Importantly, no implant failures or bleeding on probing were reported in either group at 36 months.

## 4. Discussion

The study design enabled narrow-diameter implants (NDIs) to be treated similarly to regular-diameter implants throughout the different clinical phases, including healing time and loading protocols. The study’s findings supported the acceptance of the null hypothesis, indicating that narrow dental implants have the same survival rate and clinical performance as standard diameter implants.

Both groups have been similar in relation to baseline surgical and prosthodontic variables, except in the variables of implant location and bone quality. NDIs were primarily placed in the premolar region, unlike regular-diameter implants, suggesting they were used in areas where limited bone width precluded the placement of standard-sized implants. More narrow implants were placed in bone Types I and II than wider diameter implants. This could be influenced by the fact that most implants were placed in the mandible and in the premolar regions. In a study based on cone-beam computerized tomography (CBCT), the incisor region demonstrated the highest bone density values, followed by canine and premolar sites in descending order [27]. In edentulous ridges, the bone type changes to II in the anterior maxilla, anterior mandible, and premolar mandible. In the premolar maxilla, posterior maxilla, and posterior mandible, which are the more posterior areas of the jaws, the bone type remains III [28]. The cortical bone would occupy a higher proportion of the alveolar bone in the premolar regions as compared to the molar regions, increasing its quality [28].

The reduction in the implant’s diameter is prone to several limitations, such as a smaller osseointegration surface and lower resistance to loading forces [15,16]. Furthermore, the increased ratio between the occlusal table and the narrow diameter abutments would induce a cantilever effect and increase the likelihood of mechanical fatigue and loosening [29]. Different measures can ameliorate these negative effects, like the use of intermediate original abutments and implant splinting. The use of an intermediate abutment would reduce the stress distribution to the underlying bone, favoring its stability [30,31]. Several reports have shown marginal bone stability at abutment height equal to or higher than 2.0 mm [32,33]. Additionally, the use of original abutments demonstrates superior fit and mechanical performance under cyclic loading compared to non-original configurations, potentially enhancing the long-term stability and success of the restorations [34]. Splinting multiple implants can improve their biomechanical performance by distributing lateral forces more effectively and reducing mechanical stress on individual implants [35,36,37]. These measures have been followed in this study during the prosthetic rehabilitation of the dental implants, allowing for marginal bone stability as well as implant and prosthesis survival. On the other hand, most of the implants in this study gave support to partial fixed prostheses rather than complete prostheses. In one study, implants supporting complete prostheses have shown more marginal bone loss and a lower survival rate [38]. In fixed partial protheses, lower marginal bone loss around NDIs than regular diameter implants has been reported (mean difference: −0.23 mm, 95% CI: −0.41 to −0.06, *p* = 0.01) [39].

Immediate loading has no negative influence on implant performance. This finding aligns with prior research and systematic reviews, which suggest that immediate and conventional loading protocols yield comparable marginal bone loss outcomes in standard-diameter implants [40,41,42]. The meta-analysis by Moraschini et al. [40] has revealed no significant differences in implant survival (OR 1.71, 95% CI 0.40–7.36; *p* = 0.47) or marginal bone loss (SMD −0.58, 95% CI −1.55 to 0.38; *p* = 0.24) between immediate and conventional loading. Both techniques exhibited similar rates of mechanical and biological complications. High implant survival and good marginal bone stability have also been reported by Coskunses et al. [41]. The marginal bone loss around immediately loaded NDIs has been 0.63 mm and 1.02 mm at 1- and 2-year follow-up. These good outcomes have also been confirmed by a recent meta-analysis showing no significant differences in implant survival rates between immediately loaded and conventionally loaded NDIs (Relative Risk (RR) of 0.99 (95% CI 0.94–1.03, *p* = 0.56) [42]. Additionally, marginal bone loss comparisons revealed no statistically significant difference between loading protocols (mean difference 0.03, 95% CI −0.16 to 0.23, *p* = 0.74). Immediate loading has been performed based on patient selection according to bone quality, and insertion torque could be critical for achieving successful implant-supported restorations.

Furthermore, a recent meta-analysis has assessed the use of NDIs in premolar and molar regions [43]. A high survival rate has been consistently reported, ranging from 92.73% to 100%. The pooled size effect of 0.977 (standard error: 0.004, *p* < 0.001) confirms the high survival rates of NDI across the 29 included studies. Subgroup analysis has indicated the absence of statistically significant differences in the survival rate of NDIs according to jaw type (maxilla: 97.0% [95%confidence interval (CI): 96.2% to 97.8%] and mandible: 96.5% [95% CI: 95.7% to 97.3%]) and tooth type (premolars: 97.5% [95% CI: 95.9% to 99.1%] and molars: 98.6% [95% CI: 96.5% to 99.6%]) [43]. Regarding titanium-NDIs, the study has reported a high survival rate of 98.4% (95% CI: 97.6% to 99.2%). Similarly, NDIs have similar survival rates to regular diameter implants in partial fixed prostheses (odds ratio: 0.59, 95% CI: 0.18 to 1.92, *p* = 0.38) [39]. This information aligns with the data obtained in the current clinical trial, advocating the high survival rate of NDIs. As NDIs reduce the need for alveolar ridge augmentation, they could be a good alternative to bone augmentation procedures to place regular diameter implants [11,13]. Indeed, NDIs have achieved higher patient satisfaction and lower treatment costs [20]. Moreover, the use of two-piece NDIs would allow a long-term function by offering the possibility to respond to changes in the peri-implant tissues over time [9].

Several surgical procedures are available to manage the clinical situation of horizontal alveolar bone in implant dentistry [44,45]. Sagittal osteotomy is designed to create a space by displacing the buccal cortical wall to place the dental implant [46]. Bone block grafting, alveolar ridge split, and guided bone regeneration are other surgical procedures to increase the width of the alveolar bone process [47,48]. These surgical procedures would require healing time, increased costs, graft exposure, and surgical morbidity [47,48]. Given the good NDI survival rates and the marginal bone level, these implants could be a suitable alternative to surgical bone augmentation procedures [49].

Plasma rich in growth factors has been placed in the implant alveolus before implant placement. After activation with calcium ions, a 3D scaffold of PRGF is formed at the implant surface that is rich in growth factors capable of enhancing soft and hard tissue regeneration [50,51,52]. PRGF has been shown to minimize postoperative complications [50]. These effects have impacted implant stability and osseointegration, as the use of PRGF, compared to other surgical variables, contributed to having the highest implant stability quotient at 12 weeks in a clinical study [53].

In this study, the prostheses have been free of technical complications during the 3-year follow-up. A recent systematic review with meta-analysis has shown that the implant diameter has no significant effect on the technical complications of implant-supported prostheses [39]. For implant-supported fixed dental prostheses in non-full arch cases, screw loosening emerged as the most frequent complication across different implant diameters, occurring in 17.28% of narrow, 4.08% of standard, and 12.45% of wide-diameter implants [39]. Alleviating any restriction on the selection of diameter implants and allowing the adaptation of the appropriate implant diameter to the patient’s anatomy and characteristics, and the type of prosthetic component.

This study suffers from the limitation of a non-randomized clinical trial and the short follow-up. In a randomized study design, adequate bone width to accommodate both narrow and regular-diameter implants would be a prerequisite, creating ideal conditions. Greater bone volume would be a confounding factor that may limit the extrapolation of the study’s outcomes to the use of narrow implants in non-atrophic alveolar bone. Therefore, the design of this study has allowed for a direct comparison between narrow- and regular-diameter implants based on their respective indications, closely mirroring real-world clinical scenarios. However, this type of design did not allow blinding to implant type for the researcher, but was performed in data analysis. Furthermore, full-arch cases have been very limited in this study, indicating the need for future clinical trials to validate the study outcomes in these clinical cases. Furthermore, 31% of the implants have been lost to follow-up at 3 years, which doubles the estimated drop-out for sample size calculation. The study has been affected by the COVID pandemic and its associated restrictions. This impacted the statistical power of the results. The performance of multiple imputation of missing data and the re-analysis is reported in the Supplementary File. The results were similar to those of the original data. In this study, both implant types, supporting the same prosthesis, have been compared. Data extrapolation to the clinical situation where a fixed prosthesis is supported only by NDIs is limited. Further long-term clinical trials are needed to confirm the findings of this study; however, NDIs could constitute a reliable option in restoring the alveolar ridge with horizontal atrophy.

## 5. Conclusions

Within the limitations of this study, narrow-diameter implants demonstrated comparable clinical outcomes to standard-diameter implants when supporting similar prostheses. The analysis revealed no significant differences in implant survival or incidence of technical complications between the two implant types. Further long-term clinical trials are needed to confirm the findings of this study.

## Figures and Tables

**Table 1:** Anatomical location of the dental implants included in this study.

Study Group	Location	Jaw		Total	*p*-Value
		Maxilla	Mandible		
Narrow implants	Anterior teeth	2	1	3	0.000 ^a^
	Premolars	10	21	31	
	Molars	5	19	24	
	Total	17	41	58	
Regular implants	Anterior teeth	1	1	2	
	Premolars	3	0	3	
	Molars	14	39	53	
	Total	18	40	58	
*p*-value		0.840 ^a^			

Note: ^a^ chi-square test.

**Table 2:** Description of bone type, insertion torque, and loading protocol variables for the dental implants included in the study.

Variable		Test	Control	*p*-Value
Bone type	I	6	1	0.033 ^a^
	II	37	31	
	III	14	20	
	IV	1	6	
Insertion torque (Ncm) (median; range)		60; 10 to 70	60; 10 to 65	0.888 ^b^
Loading protocol	Immediate	32	32	1.000 ^a^
	Delayed	26	26	

Note: ^a^ chi-square test; ^b^ Wilcoxon test.

**Table 3:** Prosthetic and implant performance in the study groups.

Variable		Test	Control	*p*-Value
Antagonist type	Implant	30	34	0.752 ^a^
	Tooth	27	23	
	Removable denture	1	1	
Prosthesis type	Complete	1	1	1.000 ^a^
	Partial	57	57	
Failed prosthesis	12 months	0	0	NA
	36 months	0	0	NA
Change in mean MBL-12 months (mm)	Median; range	0.0; −2.2 to 0.7	0.0; −1.0 to 1.1	0.029 ^b^
Change in mean MBL-36 months (mm)	Median; range	−0.2; −1.5 to 0.8	0.0; −1.3 to 0.8	0.079 ^c^
Implant Failure-36 months	0	0	-	
Bleeding on probing-36 months	0	0	-	

Note: ^a^ chi-square test; ^b^ Wilcoxon test; ^c^ Welch *U* test; NA: Not applicable.

## Data Availability

The raw data supporting the conclusions of this article will be made available by the authors on request.

## Supplementary Materials

The following supporting information can be downloaded at: https://www.mdpi.com/article/10.3390/dj13090420/s1. File S1: manuscript-supplementary.
