# Supplementary material for: Comparison of Narrow (<3.75 mm) and Standard (≥3.75 mm) Diameter Implants Supporting the Same Multiple Fixed Prostheses and Mirroring Real-World Clinical Scenarios: Non-Randomized Clinical Trial
Source: Dent J (Basel). 2025 Sep 12;13(9):420. doi: 10.3390/dj13090420 (PMC12468807; doi:10.3390/dj13090420)
Supplement: Supplementary file 1 [file dentistry-13-00420-s001.zip › dentistry-3774351-supplementary.pdf]

Multiple imputation of missing data of variation in marginal bone level (MBL)

Number of imputation			Mean	N	Std. Deviation	Std. Error Mean	Fraction Missing Info.
Original data	Pair 1	Variation_MBL_1 year_Test	-.2825	51	.55865	.07823	
		Variation_MBL_1 year_Control	-.0725	51	.31069	.04351	
	Pair 2	Variation_MBL_3 years_Test	-.3658	32	.46530	.08225	
		Variation_MBL_3 years_Control	-.1439	32	.41838	.07396	
1	Pair 1	Variation_MBL_1 year_Test	-.2989	58	.56415	.07408	
		Variation_MBL_1 year_Control	-.1133	58	.36263	.04762	
	Pair 2	Variation_MBL_3 years_Test	-.3217	58	.46035	.06045	
		Variation_MBL_3 years_Control	-.0780	58	.43150	.05666	
2	Pair 1	Variation_MBL_1 year_Test	-.3078	58	.55589	.07299	
		Variation_MBL_1 year_Control	-.0746	58	.32350	.04248	
	Pair 2	Variation_MBL_3 years_Test	-.3312	58	.48904	.06421	
		Variation_MBL_3 years_Control	-.1551	58	.37196	.04884	
3	Pair 1	Variation_MBL_1 year_Test	-.2972	58	.57011	.07486	
		Variation_MBL_1 year_Control	-.1200	58	.34125	.04481	
	Pair 2	Variation_MBL_3 years_Test	-.3051	58	.53035	.06964	
		Variation_MBL_3 years_Control	-.1050	58	.41187	.05408	
4	Pair 1	Variation_MBL_1 year_Test	-.3149	58	.57618	.07566	
		Variation_MBL_1 year_Control	-.1094	58	.33555	.04406	

	Pair 2	Variation_MBL_3 years_Test	-.2639	58	.50796	.06670	
		Variation_MBL_3 years_Control	-.1591	58	.40390	.05304	
5	Pair 1	Variation_MBL_1 year_Test	-.3179	58	.56977	.07481	
		Variation_MBL_1 year_Control	-.1125	58	.32512	.04269	
	Pair 2	Variation_MBL_3 years_Test	-.3994	58	.50383	.06616	
		Variation_MBL_3 years_Control	-.1044	58	.40430	.05309	
Pooled	Pair 1	Variation_MBL_1 year_Test	-.3073	58		.07517	.018
		Variation_MBL_1 year_Control	-.1059	58		.04854	.176
	Pair 2	Variation_MBL_3 years_Test	-.3243	58		.08489	.448
		Variation_MBL_3 years_Control	-.1203	58		.06579	.382

Paired Samples Statistics

Number of imputation			Relative Increase Variance	Relative Efficiency
Original data	Pair 1	Variation_MBL_1 year_Test		
		Variation_MBL_1 year_Control		
	Pair 2	Variation_MBL_3 years_Test		
		Variation_MBL_3 years_Control		
1	Pair 1	Variation_MBL_1 year_Test		
		Variation_MBL_1 year_Control		
	Pair 2	Variation_MBL_3 years_Test		
		Variation_MBL_3 years_Control		
2	Pair 1	Variation_MBL_1 year_Test		
		Variation_MBL_1 year_Control		
	Pair 2	Variation_MBL_3 years_Test		
		Variation_MBL_3 years_Control		
3	Pair 1	Variation_MBL_1 year_Test		
		Variation_MBL_1 year_Control		
	Pair 2	Variation_MBL_3 years_Test		
		Variation_MBL_3 years_Control		
4	Pair 1	Variation_MBL_1 year_Test		

		Variation_MBL_1 year_Control		
	Pair 2	Variation_MBL_3 years_Test		
		Variation_MBL_3 years_Control		
5	Pair 1	Variation_MBL_1 year_Test		
		Variation_MBL_1 year_Control		
	Pair 2	Variation_MBL_3 years_Test		
		Variation_MBL_3 years_Control		
Pooled	Pair 1	Variation_MBL_1 year_Test	.019	.996
		Variation_MBL_1 year_Control	.197	.966
	Pair 2	Variation_MBL_3 years_Test	.679	.918
		Variation_MBL_3 years_Control	.529	.929

Paired Samples Correlations

Number of imputaion			N	Correlation	Sig.
Original data	Pair 1	Variation_MBL_1 year_Test & Variation_MBL_1 year_Control	51	.130	.364
	Pair 2	Variation_MBL_3 years_Test & Variation_MBL_3 years_Control	32	-.167	.362
1	Pair 1	Variation_MBL_1 year_Test & Variation_MBL_1 year_Control	58	.078	.559
	Pair 2	Variation_MBL_3 years_Test & Variation_MBL_3 years_Control	58	-.137	.304
2	Pair 1	Variation_MBL_1 year_Test & Variation_MBL_1 year_Control	58	.046	.731
	Pair 2	Variation_MBL_3 years_Test & Variation_MBL_3 years_Control	58	-.107	.424
3	Pair 1	Variation_MBL_1 year_Test & Variation_MBL_1 year_Control	58	.160	.231

	Pair 2	Variation_MBL_3 years_Test & Variation_MBL_3 years_Control	58	.098	.464
4	Pair 1	Variation_MBL_1 year_Test & Variation_MBL_1 year_Control	58	.170	.203
	Pair 2	Variation_MBL_3 years_Test & Variation_MBL_3 years_Control	58	-.129	.333
5	Pair 1	Variation_MBL_1 year_Test & Variation_MBL_1 year_Control	58	.236	.075
	Pair 2	Variation_MBL_3 years_Test & Variation_MBL_3 years_Control	58	-.244	.065
Pooled	Pair 1	Variation_MBL_1 year_Test & Variation_MBL_1 year_Control	58	.139	
	Pair 2	Variation_MBL_3 years_Test & Variation_MBL_3 years_Control	58	-.105	

Number of imputation			Paired Differences				
			Mean	Std. Deviation	Std. Error Mean	95% Confidence Interval of the Difference	
						Lower	Upper
Original data	Pair 1	Variation_MBL_1 year_Test & Variation_MBL_1 year_Control	-.21000	.60296	.08443	-.37958	-.04042
	Pair 2	Variation_MBL_3 years_Test & Variation_MBL_3 years_Control	-.22187	.67560	.11943	-.46545	.02170

1	Pair 1	Variation_MBL_1 year_Test & Variation_MBL_1 year_Control	-.18562	.64633	.08487	-.35557	-.01568
	Pair 2	Variation_MBL_3 years_Test & Variation_MBL_3 years_Control	-.24375	.67281	.08834	-.42066	-.06684
2	Pair 1	Variation_MBL_1 year_Test & Variation_MBL_1 year_Control	-.23329	.63012	.08274	-.39897	-.06760
	Pair 2	Variation_MBL_3 years_Test & Variation_MBL_3 years_Control	-.17609	.64529	.08473	-.34576	-.00642
3	Pair 1	Variation_MBL_1 year_Test & Variation_MBL_1 year_Control	-.17719	.61586	.08087	-.33912	-.01526
	Pair 2	Variation_MBL_3 years_Test & Variation_MBL_3 years_Control	-.20011	.63877	.08387	-.36806	-.03215
4	Pair 1	Variation_MBL_1 year_Test & Variation_MBL_1 year_Control	-.20550	.61565	.08084	-.36737	-.04362
	Pair 2	Variation_MBL_3 years_Test & Variation_MBL_3 years_Control	-.10478	.68865	.09042	-.28585	.07629
5	Pair 1	Variation_MBL_1 year_Test & Variation_MBL_1 year_Control	-.20539	.58565	.07690	-.35938	-.05140

	Pair 2	Variation_MBL_3 years_Test & Variation_MBL_3 years_Control	-.29500	.71887	.09439	-.48402	-.10598
Pooled	Pair 1	Variation_MBL_1 year_Test & Variation_MBL_1 year_Control	-.20140		.08469	-.36770	-.03509
	Pair 2	Variation_MBL_3 years_Test & Variation_MBL_3 years_Control	-.20395		.11820	-.45001	.04212

Number of imputation			t	df	Sig. (2-tailed)	Fraction Missing Info.	Relative Increase Variance
Original data	Pair 1	Variation_MBL_1 year_Test & Variation_MBL_1 year_Control	-2.487	50	.016		
	Pair 2	Variation_MBL_3 years_Test & Variation_MBL_3 years_Control	-1.858	31	.073		
1	Pair 1	Variation_MBL_1 year_Test & Variation_MBL_1 year_Control	-2.187	57	.033		
	Pair 2	Variation_MBL_3 years_Test & Variation_MBL_3 years_Control	-2.759	57	.008		
2	Pair 1	Variation_MBL_1 year_Test & Variation_MBL_1 year_Control	-2.820	57	.007		
	Pair 2	Variation_MBL_3 years_Test & Variation_MBL_3 years_Control	-2.078	57	.042		

3	Pair 1	Variation_MBL_1 year_Test & Variation_MBL_1 year_Control	-2.191	57	.033		
	Pair 2	Variation_MBL_3 years_Test & Variation_MBL_3 years_Control	-2.386	57	.020		
4	Pair 1	Variation_MBL_1 year_Test & Variation_MBL_1 year_Control	-2.542	57	.014		
	Pair 2	Variation_MBL_3 years_Test & Variation_MBL_3 years_Control	-1.159	57	.251		
5	Pair 1	Variation_MBL_1 year_Test & Variation_MBL_1 year_Control	-2.671	57	.010		
	Pair 2	Variation_MBL_3 years_Test & Variation_MBL_3 years_Control	-3.125	57	.003		
Pooled	Pair 1	Variation_MBL_1 year_Test & Variation_MBL_1 year_Control	-2.378	644	.018	.082	.086
	Pair 2	Variation_MBL_3 years_Test & Variation_MBL_3 years_Control	-1.725	21	.099	.488	.786

Paired Samples Test

Number of imputation			Relative Efficiency
Original data	Pair 1	Variation_MBL_1 year_Test & Variation_MBL_1 year_Control	
	Pair 2	Variation_MBL_3 years_Test & Variation_MBL_3 years_Control	
1	Pair 1	Variation_MBL_1 year_Test & Variation_MBL_1 year_Control	

	Pair 2	Variation_MBL_3 years_Test & Variation_MBL_3 years_Control	
2	Pair 1	Variation_MBL_1 year_Test & Variation_MBL_1 year_Control	
	Pair 2	Variation_MBL_3 years_Test & Variation_MBL_3 years_Control	
3	Pair 1	Variation_MBL_1 year_Test & Variation_MBL_1 year_Control	
	Pair 2	Variation_MBL_3 years_Test & Variation_MBL_3 years_Control	
4	Pair 1	Variation_MBL_1 year_Test & Variation_MBL_1 year_Control	
	Pair 2	Variation_MBL_3 years_Test & Variation_MBL_3 years_Control	
5	Pair 1	Variation_MBL_1 year_Test & Variation_MBL_1 year_Control	
	Pair 2	Variation_MBL_3 years_Test & Variation_MBL_3 years_Control	
Pooled	Pair 1	Variation_MBL_1 year_Test & Variation_MBL_1 year_Control	.984
	Pair 2	Variation_MBL_3 years_Test & Variation_MBL_3 years_Control	.911
